# Redundancy, noise, and plasticity: repetitive DNA as an epigenetic intelligence backbone of inflammatory regulation

**DOI:** 10.1093/eep/dvaf028

**Published:** 2025-10-22

**Authors:** Valentina Bollati, Elia Biganzoli

**Affiliations:** EPIGET Lab -Department of Clinical Sciences and Community Health (DISCCO), “Dipartimento di Eccellenza 2023–2027”, Università degli Studi di Milano, 20122 Milan, Italy; INES (Institute of Epigenetics for Smiles), Università degli Studi di Milano, 20157 Milan, Italy; INES (Institute of Epigenetics for Smiles), Università degli Studi di Milano, 20157 Milan, Italy; Unit of Medical Statistics, Bioinformatics and Epidemiology, Department of Biomedical and Clinical Sciences (DIBIC), Università degli Studi di Milano, 20157 Milan, Italy

**Keywords:** repetitive DNA, LINE1, epigenetic intelligence, redundancy, noise, plasticity

## Abstract

Background: Repetitive DNA elements such as LINE1 have been proposed to support a redundant distributed, adaptive layer of gene regulation, contributing to “Epigenetic Intelligence” (EI). However, empirical evidence for their role in modulating inflammatory responses to environmental exposures remains limited. Objectives: We investigated whether the association of PM with an aerodynamic diameter ≤10 µm (PM_10_) with fibrinogen levels is modulated by LINE1 methylation (effect modification), acting as an epigenetic buffer of systemic inflammation in response to air pollution, looking at the EI hypothesis. Methods: We analyzed data from the SPHERE cohort (*n* = 1630), a population-based study in Northern Italy. Daily residential exposure to PM₁₀ was estimated, and LINE1 methylation was assessed via pyrosequencing. Fibrinogen was used as a biomarker of systemic inflammation. Generalized Additive Models with tensor product interactions were used to evaluate the PM₁₀ × LINE1 interaction, adjusting for relevant confounders. Results: The interaction between PM₁₀ exposure and LINE1 methylation was statistically supported (EDF ≈ 4.45, *P* < 0.001), with the model explaining ∼33% of deviance (adj. R² = 0.39). Individuals in the lowest tertile of LINE1 methylation exhibited a stronger positive association between PM₁₀ and fibrinogen, whereas those in the highest tertile showed a blunted response, suggesting a buffering modification effect. Results were confirmed by MARS models. Conclusions: Our findings were coherent with the concept of EI. LINE1 methylation modulates the inflammatory response to environmental stressors, possibly acting as an adaptive epigenetic filter that buffers weak or transient signals. This distributed regulatory capacity may be critical for immune homeostasis under the dynamic environmental challenge.

## Introduction

The regulation of gene expression is not merely a binary process of activation or repression, but rather a complex, adaptive system that integrates molecular cues, environmental inputs, and epigenetic memory. While classical models of epigenetic control emphasize promoter methylation, histone modifications, and transcription factor binding at gene-specific loci, a growing body of evidence suggests that non-coding and repetitive DNA elements contribute to a distributed layer of regulatory flexibility and robustness [1–4].

In previous work, we introduced the concept of Epigenetic Intelligence (EI), a theoretical model that frames the epigenome as a multilayered computational system capable of learning, buffering, and adapting to external stimuli [5]. EI consists of two interacting regulatory layers: (i) The symbolic layer, composed of canonical gene-specific mechanisms (e.g. promoter methylation, histone acetylation), which operates via rule-based, discrete logic akin to symbolic artificial intelligence [5, 6]. This layer governs precise transcriptional outcomes in response to defined inputs; (ii) the subsymbolic layer, on the other hand, is constituted by repetitive elements such as LINEs, SINEs, and endogenous retroviruses. These elements function as a probabilistic, distributed network with redundant nodes that modulates chromatin topology, filters transcriptional noise, and supports long-term regulatory plasticity [7–9].

This neuro-symbolic architecture parallels models in artificial intelligence, where symbolic reasoning modules interact with distributed neural networks to generate context-sensitive, adaptive behavior [5, 6]. Similarly, we propose that the genome integrates symbolic control and subsymbolic modulation to orchestrate physiological responses to complex environmental stimuli.

A paradigmatic domain where this dual-layered architecture is especially relevant is inflammation. The immune system must rapidly detect and neutralize threats while avoiding overactivation that could damage host tissues and organs. This requires not only sharp responsiveness but also graded, noise-resistant modulation across a spectrum of stimuli. Canonical pathways such as NF-κB [2, 10], MAPKs, and JAK-STAT coordinate these responses, but their outputs are shaped by epigenetic context, including chromatin accessibility, histone marks, and DNA methylation [11].

Acute inflammation involves rapid activation of cytokine genes such as TNF-α, IL-6, and IL-1β, typically located in regions primed by active histone marks and low DNA methylation [12]. Repeated or chronic stimulation can induce long-lasting epigenetic reprogramming, as observed in trained immunity, where innate immune cells retain a memory of past exposures via histone modification and enhancer remodeling [13, 14]. Conversely, the resolution of inflammation involves silencing of pro-inflammatory loci and activation of anti-inflammatory genes like IL10, often via chromatin closing or bivalent marks [11]. Dysregulation of these processes is implicated in autoimmunity, chronic low-grade inflammation, and inflammaging [15].

Yet the role of repetitive DNA in this process remains underexplored. These elements may contribute to regulatory redundancy, distributing pro-inflammatory signals across the genome to prevent over-reliance on single loci; noise filtering, by maintaining repressive chromatin states that dampen weak or transient inputs; and plasticity, allowing adaptive reconfiguration of enhancer networks in response to chronic stress or repeated exposure [7–9].

We hypothesize that this subsymbolic layer acts as a buffering system, enhancing both stability and adaptability of inflammatory gene regulation, in an optimal stochastic control perspective. A failure in this system, through hypomethylation, chromatin loosening, or transposon activation, may underlie phenomena such as chronic inflammation, immune memory dysregulation, and inflammaging [15–18].

Previous analyses in the SPHERE cohort [19] have shown that short-term exposure to airborne particulate matter (PM₁₀) is associated with increased circulating levels of fibrinogen, a key acute-phase protein and systemic biomarker of inflammation [20]. Elevated fibrinogen reflects endothelial activation, enhanced coagulation, and low-grade inflammatory signaling, and has been linked to heightened risk of cardiovascular and respiratory disease. However, considerable interindividual variability remains unexplained, coherently with the action of latent buffering mechanisms that modulate inflammatory sensitivity to environmental factors.

To investigate this hypothesis empirically, we focus on LINE1 elements, a class of long interspersed nuclear elements widely used as a surrogate marker of global repetitive DNA methylation [21]. We evaluate whether LINE1 methylation modulates the inflammatory response to PM₁₀, specifically its interaction with fibrinogen, in a population-based molecular epidemiology study.

**Figure 1.**
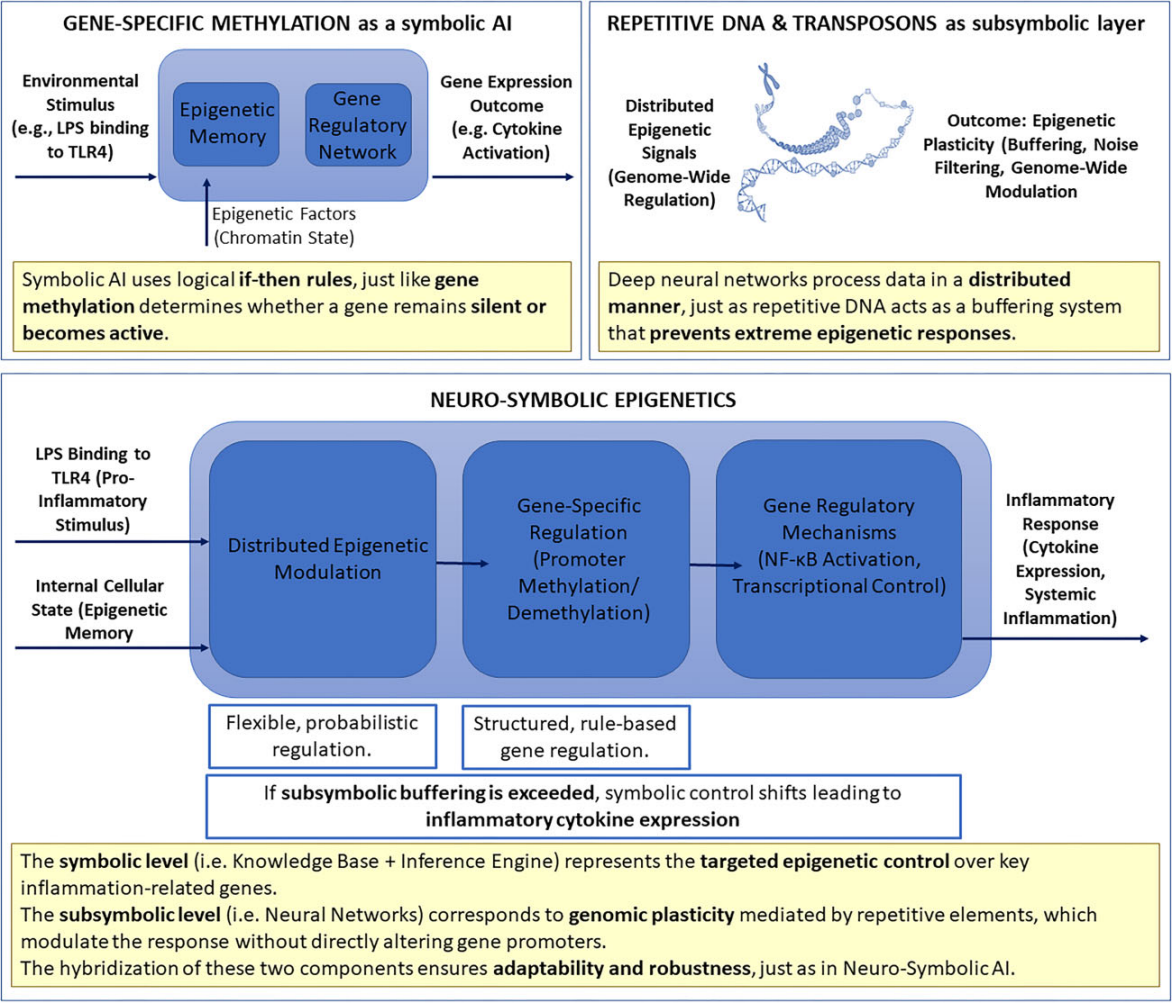
Neuro-symbolic epigenetics: a hybrid model of gene regulation in inflammation. The symbolic layer (top left) represents gene-specific epigenetic control, such as promoter methylation, which functions through rule-based mechanisms similar to symbolic AI. This layer determines whether inflammation-related genes are turned on or off in response to specific stimuli (e.g. LPS via TLR4). The subsymbolic layer (top right) consists of repetitive DNA and transposons, which contribute to genome-wide modulation through distributed, probabilistic processes. This layer acts as an epigenetic buffer, filtering noise and enabling plasticity. The integrated neuro-symbolic framework (bottom panel) illustrates how internal cellular states and external inflammatory signals are processed through subsymbolic modulation and symbolic gene-specific mechanisms. Together, these layers determine the activation of transcriptional programs (e.g. NF-κB-mediated cytokine expression). If the buffering capacity of the subsymbolic layer is exceeded, the symbolic system activates inflammatory outputs. This hybrid architecture ensures both adaptability and robustness, mirroring principles of neuro-symbolic artificial intelligence.

This study represents a proof-of-concept (Fig. 1) for the EI framework, providing empirical grounding to a theoretical model that links genome architecture, environmental responsiveness, and immune regulation.

## Results

### Participant characteristics

A total of 1630 participants from the SPHERE cohort were included in this study. All subjects were overweight or obese (BMI ≥ 25 kg/m²), following the inclusion criteria. Descriptive statistics for demographic, clinical, and exposure variables have been previously reported in [20]. LINE-1 methylation (logit) showed a mean of 1.08 (SD = 0.13) across participants. The median PM₁₀ exposure (lag 0–1) was 33.0 µg/m³, with an interquartile range of 22.0–52.0 µg/m³, indicating substantial day-to-day variability. Circulating fibrinogen levels averaged 331.68 mg/dL (SD = 63.82).

### Interaction between PM_10_ exposure and LINE-1 methylation on fibrinogen levels

We tested the hypothesis that LINE-1 methylation modulates the relationship between PM₁₀ exposure and systemic inflammation, as measured by fibrinogen. In models without interaction terms, higher PM₁₀ concentrations were positively associated with fibrinogen levels (β = 0.14, *P*-value = 0.0019), indicating that greater short-term PM₁₀ exposure was related to higher systemic inflammation. Using Generalized Additive Models (GAMs) with a tensor product smooth interaction term, we observed the evidence of the interaction between PM₁₀ (lag 0–1) and LINE-1 methylation on fibrinogen levels (F = 5.8, *P* = 0.003), indicating that the effect of PM₁₀ varies as a function of LINE-1 methylation.

**Figure 2.**
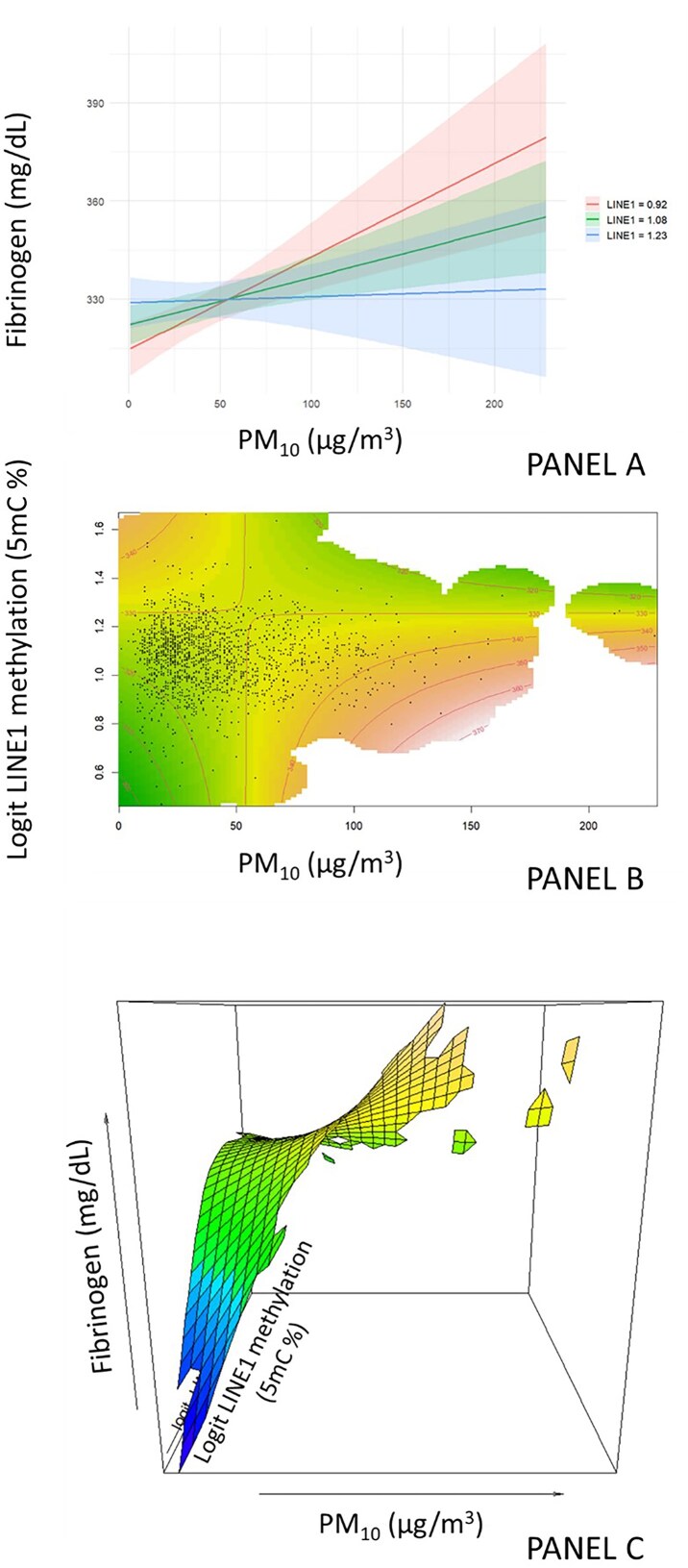
Interaction between PM₁₀ and LINE1 methylation in predicting fibrinogen levels through a generalized additive model. (A) Predicted dose–response curves of fibrinogen (*y*-axis) for three strata of LINE1 methylation (logit-transformed): lowest tertile (T1, hypomethylated), middle tertile (T2), and highest tertile (T3, hypermethylated). Individuals in T1 exhibit a steeper increase in fibrinogen with rising PM₁₀, whereas T3 shows an attenuated response. (B) Contour plot of the tensor interaction smooth from the GAM: isolines represent fitted fibrinogen levels across the two-dimensional space defined by PM₁₀ (x-axis) and LINE1 methylation (*y*-axis). Regions with higher predicted fibrinogen correspond to low LINE1 and high PM₁₀, highlighting the buffering effect of methylation. (C) Three-dimensional surface plot of the same GAM-predicted interaction, showing fibrinogen (mg/dL, *z*-axis) as a function of PM₁₀ exposure (µg/m³, *x*-axis) and LINE1 methylation (logit scale, *y*-axis). The perspective highlights the non-linear buffering effect of higher LINE1 methylation on PM₁₀-associated inflammation.

As illustrated in Fig. 2, higher LINE-1 methylation was associated with blunted fibrinogen responses to PM₁₀, suggesting a possible buffering effect. In contrast, lower LINE-1 methylation values displayed a steeper positive association between PM₁₀ and fibrinogen.

This pattern is visually detailed in the stratified dose–response curves of Fig. 2 panel A, where the relationship between PM₁₀ exposure and fibrinogen levels varies markedly across LINE-1 methylation tertiles. Specifically, individuals in the lowest tertile of LINE-1 methylation display a pronounced increase in fibrinogen with rising PM₁₀, consistent with heightened inflammatory reactivity. In contrast, participants in the highest tertile show a substantially flatter slope, indicating reduced sensitivity to the same environmental stimulus. The buffering effect is further supported by the contour plot in Fig. 2 panel B, where regions of high PM₁₀ and low LINE-1 coincide with the highest predicted fibrinogen levels. Conversely, at equivalent PM₁₀ concentrations, individuals with higher LINE-1 methylation fall within a lower predicted fibrinogen range, suggesting that LINE-1 methylation may modulate the translation of environmental exposure into systemic inflammatory output. Finally, the 3D surface plot (Fig. 2 panel C) reinforces the non-linear nature of this interaction, illustrating a sharp fibrinogen gradient emerging only in the context of low methylation. In contrast, the surface flattens in the hypermethylated spectrum. Together, these visualizations provide convergent evidence coherently with a modulatory, non-additive role of repetitive DNA methylation in inflammatory responsiveness.

To aid interpretation, we estimated the change in fibrinogen concentration associated with a 10 µg/m³ increase in PM₁₀ at representative LINE-1 methylation levels. At the 10th percentile of LINE-1 methylation, PM₁₀ was associated with a 2.85 g/l (3.90–1.79), whereas in the high LINE-1 methylation group (90th percentile) the corresponding change was 0.182 (C.I. 1.11–0.742) g/l. These differences are consistent with a buffering effect of higher LINE-1 methylation on PM₁₀-induced inflammation.

The interaction remained robust after adjusting for a range of covariates, including age, sex/menopausal status, BMI, smoking status, monocyte count, and C-reactive protein (CRP) levels. The final GAM model explained approximately 32.8% of the variability in fibrinogen levels (adjusted R² = 0.39). Additional adjustment for season and meteorological parameters (apparent temperature) did not materially alter the findings. Additional adjustment for season and apparent temperature yielded results similar to the main models (Supplementary Table S1).

### Alternative modeling: MARS approach

To complement the GAM analysis, we applied Multivariate Adaptive Regression Splines (MARS) to detect data-driven non-linear and interaction effects. The MARS model identified an interaction between PM₁₀ and LINE-1 methylation as one of the top predictors of fibrinogen variability. Specifically, the term involving the interaction between PM₁₀ and LINE-1 methylation was selected in 5 model subsets and accounted for 19.2% of GCV-based importance and 24.0% of RSS-based importance, underscoring its substantial contribution to model performance.

The final MARS model explained ∼42.2% of the variability in fibrinogen levels (R² = 0.42; GCV = 2451.5; Akaike Information Criterion = 16 220), with 11 terms selected out of 18 and 7 predictors retained. Several high-order interaction terms were also included, such as those between CRP, BMI, and LINE-1 methylation, further supporting the hypothesis of non-linear, synergistic modulation of inflammation by epigenetic and environmental factors. These results are consistent with and reinforce the findings obtained through GAM modeling, providing convergent evidence for a modulatory role of LINE-1 methylation in shaping inflammatory responses to PM₁₀.

## Discussion

In this study, we provide empirical evidence that repetitive DNA methylation, specifically LINE-1 methylation, modulates individual inflammatory sensitivity to airborne PM₁₀. The association between short-term PM₁₀ exposure and fibrinogen levels, a biomarker of systemic inflammation, varied according to LINE-1 methylation status: participants with lower methylation displayed a steeper increase in fibrinogen, whereas those with higher methylation showed a more attenuated response. These results are consistent with the EI framework, which posits that repetitive DNA acts as a subsymbolic regulatory layer capable of buffering environmental signals.

In interpreting these findings, it is important to distinguish between empirical results and broader conceptual considerations. The data-driven contribution of this work is the demonstration, in a well-characterized population, of a statistically significant interaction between PM₁₀ and LINE-1 methylation on fibrinogen levels, robust across two independent modeling approaches and adjusted for relevant covariates. The subsequent discussion situates these results within the EI framework and considers potential implications for inflammatory regulation, trained immunity, and lifestyle interventions. These latter points are presented as speculative extensions intended to guide future research, rather than as conclusions directly supported by the present dataset.

Repetitive elements such as LINE-1 have long been regarded as genomic “noise” or “junk,” but emerging literature suggests they may contribute to maintaining genome stability, regulating chromatin accessibility, and shaping transcriptional responsiveness [1–3]. While our results are consistent with the possibility that methylation of repetitive elements serves a buffering function limiting inflammatory overactivation in response to acute environmental insults, we did not directly assess chromatin structure, histone modifications, or transposon activity in this study. Therefore, these proposed functions should be interpreted as theoretical extensions of our empirical findings.

From the perspective of the EI model, the genome may include not only gene-specific logic gates (the “symbolic” layer) but also a distributed, probabilistic “subsymbolic” network composed of repetitive sequences. In this architecture, the symbolic layer governs targeted gene expression (e.g. cytokine induction via NF-κB), while the subsymbolic layer could, in principle, modulate global chromatin dynamics and noise filtering. Although our observation that higher LINE-1 methylation correlates with a dampened fibrinogen response is compatible with this model, direct mechanistic evidence is not available in the present dataset. Future studies using ATAC-seq, ChIP-seq, or single-cell methylome profiling will be required to determine whether such chromatin-based buffering mechanisms operate in humans.

Within this framework, LINE-1 methylation is not a fixed trait but the cumulative outcome of multiple environmental and endogenous influences, the integrated exposome. Acute PM₁₀ exposure contributes to shaping LINE-1 methylation, but its direct impact is relatively small compared to other factors such as diet, psychosocial stress, chronic infections, and co-exposure to other pollutants. The methylation level observed at a given time therefore represents an environmentally shaped baseline that modulates the inflammatory response to acute PM₁₀ challenges. In this sense, LINE-1 operates primarily as a systemic effect modifier, buffering or amplifying the fibrinogen response according to its set point, rather than as a direct mediator of PM₁₀ effects. While mediation pathways cannot be entirely excluded, the current evidence and our cross-sectional design support interpreting LINE-1 chiefly as an adaptive epigenetic filter that has been tuned by cumulative exposures over the life course.

Our findings support a threefold model for how repetitive DNA may buffer inflammatory responses:

(1) Redundancy-based buffering: Repetitive elements are widely distributed across the genome and often contain binding motifs for transcription factors such as NF-κB, a master regulator of the inflammatory response [2, 10]. Rather than modulating gene expression at isolated loci, these sequences provide a regulatory network capable of distributing the effects of pro-inflammatory stimuli across multiple genomic regions [22].

In this framework, redundancy acts as a stabilizing force. If a single locus becomes epigenetically altered, for example, through demethylation or chromatin remodeling, other repetitive elements with similar regulatory features can maintain the overall chromatin state. This minimizes the risk of abrupt or exaggerated transcriptional responses [3].

As an additional example in the context of LPS-induced NF-κB activation, this redundancy may ensure that pro-inflammatory gene activation unfolds in a graded and distributed manner, rather than as an all-or-nothing switch. NF-κB may interact not only with canonical gene promoters but also with repetitive sequences that modulate chromatin accessibility at multiple sites simultaneously [23]. This distributed architecture could explain how immune responses scale proportionally to stimulus strength, reducing the risk of runaway inflammation [24];

(2) Epigenetic noise filtering: Beyond redundancy, repetitive elements contribute to the reduction of transcriptional noise, particularly in response to weak or transient environmental signals. These elements are often heavily methylated, contributing to heterochromatin formation and the silencing of nearby genomic regions [25, 26]. This widespread methylation functions as a filter, preventing sporadic or low-intensity stimuli from inappropriately activating inflammatory genes [27]. This noise-buffering mechanism ensures that only sustained or high-intensity stimuli lead to significant epigenetic remodeling and gene expression. For example, demethylation of the TNF-α promoter is typically observed only after prolonged or repeated LPS exposure [28]. Repetitive elements in the vicinity may absorb minor fluctuations in NF-κB activity [29], preventing premature transcriptional activation of cytokines [30].
(3) Dynamic plasticity: Although not directly tested here, the observation that individuals with lower LINE-1 methylation exhibit a heightened inflammatory response suggests a potential for epigenomic destabilization under chronic environmental stress. Transposable elements, which are often epigenetically silenced in normal conditions, can become reactivated in response to environmental pressure. This reactivation may lead to novel insertion events, introducing new enhancer elements or disrupting existing regulatory domains.

Such epigenetic plasticity allows the genome to reconfigure regulatory networks, enabling long-term adaptation to inflammatory stress. While this mechanism may enhance immune efficiency in the short term, it also carries risks. Aberrant activation of transposons can lead to persistent changes in cytokine regulation, potentially contributing to chronic inflammatory states or autoimmune pathogenesis.

In this sense, transposable elements function analogously to subsymbolic learning in neural networks, where weight distributions shift in response to repeated inputs. Just as subsymbolic AI systems adapt through iterative tuning, the epigenome responds to chronic stress by reconfiguring its regulatory architecture, sometimes adaptively, and sometimes with pathological consequences.

The buffering capacity of repetitive DNA elements may represent a critical layer of immune regulation, ensuring both responsiveness and restraint in the face of environmental challenges. Disruption of this balance, through epigenetic instability, loss of methylation, or inappropriate activation of transposable elements, may contribute to a range of chronic inflammatory conditions [3, 31].

A failure in redundancy-based buffering may result in hyperactivation of specific inflammatory loci, especially if NF-κB binding becomes concentrated on a limited number of exposed sites. Without the distributed regulation normally conferred by repetitive elements, even moderate stimuli could provoke disproportionate transcriptional responses, fueling chronic inflammation and tissue damage [32, 33]. Similarly, compromised noise-reduction mechanisms may allow transient or low-level signals to inappropriately trigger cytokine expression. This could help explain how individuals repeatedly exposed to low-grade environmental stressors (e.g. pollution, psychosocial stress), in the absence of compensatory interventions, develop low-grade chronic inflammation, a key feature of conditions such as cardiometabolic disease, neurodegeneration, and autoimmune disorders [15, 16].

The dynamic plasticity of repetitive elements also intersects with the concept of trained immunity, in which innate immune cells retain a memory of past exposures. Epigenetic reprogramming through histone modifications and enhancer remodeling has been implicated in this phenomenon [14]. We propose that repetitive DNA contributes to this adaptability by facilitating enhancer rewiring through transposon-derived sequences [22]. Conversely, when this plasticity becomes dysregulated, due to persistent stimuli or failure of repression mechanisms, it may promote inflammaging, a chronic, low-grade inflammatory state associated with aging and immune senescence [34, 35]. In this view, EI provides a framework to reconcile the apparent contradiction between immune memory and immune dysregulation. At present, there is no empirical evidence to define a precise threshold of intensity, duration, or stimulus type at which this “memory” function shifts from protective to detrimental. This remains a speculative, yet potentially important, aspect for future investigation.

Emerging evidence suggests that lifestyle interventions, such as dietary modulation, physical activity, sleep hygiene, stress reduction, and exposure to natural environments, may influence the epigenetic landscape of repetitive elements, potentially enhancing their buffering capacity. Several studies have shown that caloric restriction, intermittent fasting, and diets rich in polyphenols (e.g. resveratrol, curcumin) can increase global DNA methylation stability and preserve heterochromatin integrity, particularly in regions enriched with LINE-1 and Alu elements [36, 1, 2]. These modifications may reinforce the silencing of transposable elements, limiting their inappropriate activation under stress.

Physical exercise, especially endurance and moderate-intensity aerobic activity, has been associated with decreased inflammation and changes in repetitive element methylation in blood cells, suggesting a systemic effect on chromatin dynamics [37, 38]. Similarly, psychological stress has been linked to demethylation of repetitive sequences and increased transcriptional noise, whereas mindfulness-based interventions have been shown to stabilize DNA methylation patterns, possibly restoring epigenetic control over immune-related loci [39–41].

Furthermore, circadian alignment, adequate sleep, and exposure to natural light may entrain epigenetic rhythms and reduce the epigenetic drift of repetitive regions over time. These elements of lifestyle not only modulate hormonal and immune function but also impact the epigenetic resilience of the genome to inflammatory stimuli [42–44].

Taken together, these observations support the idea that repetitive elements are not only passive sensors of environmental inputs, but also active substrates of behavioral and lifestyle modulation. Enhancing the stability, silencing, and adaptive plasticity of these regions through non-pharmacological means could represent a novel, systemic approach to promoting immune resilience and preventing chronic inflammatory diseases.

## Strengths and limitations

Our study benefits from a well-characterized, population-based cohort with harmonized exposure data, biomarker measurement, and epigenetic profiling. The use of both GAM and MARS models provides convergent evidence for the moderating role of LINE-1 methylation, supporting the robustness of the interaction observed.

We also focused on fibrinogen, a clinically validated acute-phase protein with established associations to PM exposure and cardiovascular outcomes. This outcome variable allows us to link theoretical models of genome architecture with tangible markers of physiological inflammation.

Several limitations must also be acknowledged. First, our study is cross-sectional, limiting causal inference. Although temporality is preserved in the short-term exposure design, longitudinal data would strengthen claims about adaptive or maladaptive epigenetic plasticity. Second, LINE-1 methylation does not capture the full complexity of repetitive DNA regulation, including histone modifications, non-CpG methylation, or expression of transposable elements. Moreover, the LINE-1 methylation levels measured in this study represent an average across consensus sequences, masking the variability that may exist between individual LINE-1 copies located in different genomic contexts. Future studies should aim to dissect this heterogeneity by investigating specific repetitive elements, ideally using long-read sequencing technologies capable of resolving methylation patterns at single-locus resolution across the repetitive genome. Third, the generalizability of our findings may be limited by the demographic and clinical profile of the SPHERE cohort, which consists predominantly of middle-aged to older adults with a high prevalence of overweight and obesity. However, because BMI is an established determinant of LINE-1 methylation, its influence on inflammatory and epigenetic patterns is likely incorporated into the observed effect modification, partially mitigating concerns about extrapolation to populations with different BMI distributions. Additionally, we selected PM₁₀ as the exposure metric because high-quality daily measurements with complete temporal coverage were available for the entire study period from the regional environmental protection agency (ARPA Lombardia), whereas PM₂.₅ data were not consistently available for all participants, which would have markedly reduced sample size and statistical power. Importantly, in our previous work on the same population, we observed significant associations between short-term PM₁₀ exposure and multiple biological endpoints, including extracellular vesicle release and miRNA expression, supporting the relevance of PM₁₀ as a metric for investigating inflammatory and coagulation pathways in this cohort. While the EI framework proposes that chronic environmental stress can shape and eventually erode repetitive element plasticity, this chronic dimension is not directly addressed in our dataset and should be considered a conceptual extension. Future research incorporating repeated methylation measurements and longer-term exposure assessments will be essential to test this aspect of the model. Moreover, although our models adjusted for multiple relevant covariates, we cannot exclude residual confounding from unmeasured factors such as socioeconomic status, dietary patterns, microbiome composition, or psychosocial stress. These factors may influence both LINE-1 methylation and fibrinogen levels through behavioral, metabolic, or inflammatory pathways. For example, lower socioeconomic status has been linked to chronic low-grade inflammation and altered DNA methylation profiles, while antioxidant-rich diets have been associated with higher global methylation and lower fibrinogen. Such influences could contribute to the baseline variability of LINE-1 methylation, which itself modulates the fibrinogen response to PM₁₀. Lastly, our study focused on fibrinogen as the sole marker of systemic inflammation. This choice was motivated by its established clinical relevance and prior evidence of association with PM exposure in the same population. However, this approach does not encompass the broader spectrum of inflammatory and neuroendocrine responses to air pollution. Other biomarkers, such as pro-inflammatory cytokines (e.g. TNF-α) and stress-related hormones (e.g. cortisol), have been linked to particulate matter exposure and shown to interact with epigenetic regulation [45]. Future studies integrating multiple biomarkers could provide a more comprehensive understanding of the mechanisms by which air pollution influences systemic inflammation and stress responses.

In conclusion, in our population-based setting, the patterns we observe are more consistent with physiological regulatory responses than with pathological destabilization. The modulation of LINE-1 methylation appears to function as a buffering mechanism, attenuating inflammatory responsiveness under chronic, environmentally relevant exposures. While acute, above-threshold insults such as ionizing radiation or high-dose toxicants may indeed trigger rapid and more extreme hypomethylation of repetitive elements, our data suggest that in real-world conditions LINE-1 primarily acts within a physiological adaptive range.

Our findings, in fact, support the hypothesis that repetitive DNA methylation, particularly LINE-1 methylation, modulates systemic inflammatory responses to environmental pollutants. This effect is consistent with the EI framework, in which the genome integrates symbolic (gene-specific) and subsymbolic (repetitive) layers to buffer and adapt to environmental signals. LINE-1 methylation may serve not only as a marker of global epigenetic status, but as a functional mediator of inflammatory resilience. These results advance our understanding of how genome architecture contributes to environmental sensitivity and offer a conceptual bridge between molecular epidemiology and systems epigenetics.

## Materials and methods

### Study population

Participants were recruited as part of the SPHERE study (Susceptibility to Particle Health Effects, miRNAs and Exosomes), a cross-sectional molecular epidemiology study based in Northern Italy [19]. Recruitment occurred between September 2010 and March 2015 at the Center for Obesity and Work Department of Preventive Medicine, IRCCS Fondazione Ca’ Granda—Ospedale Maggiore Policlinico, Milan, Italy.

Eligible participants were adults (≥18 years) who met the following criteria: (i) overweight or obese, defined as body mass index (BMI) between 25 and 30 kg/m² or ≥ 30 kg/m², respectively; (ii) resident in the Lombardy region at the time of recruitment; (iii) willing to provide written informed consent and to donate blood and urine samples. Exclusion criteria included: a history of cancer, cardiovascular events (heart disease or stroke) in the previous year, and major chronic neurological or psychiatric conditions (e.g. multiple sclerosis, Alzheimer’s disease, Parkinson’s disease, depression, bipolar disorder, schizophrenia, epilepsy).

The participation rate among invited subjects was 92%. All participants signed an informed consent form, and the study protocol was approved by the institutional ethics committee (approval no. 1425), following the Declaration of Helsinki.

Quantitative determination of fibrinogen in citrate plasma samples was obtained on the automated I.L. Coagulation System (Instrumentation Laboratory S.p.A., Milan, Italy).

Further details on the study design and recruitment procedures have been published previously (19).

PM₁₀ exposure was assigned to each participant based on the fixed-site monitoring station closest to their residential address. As described in [19], in case of incomplete daily series for a given monitor, missing values were imputed using an algorithm that integrates the annual average of the incomplete series with PM₁₀ concentrations from the nearest and most highly correlated monitors. Only participants with complete data for LINE-1 methylation, fibrinogen, and covariates were retained for analysis (complete-case approach). LINE-1 methylation and fibrinogen measurements were available for 97% and 99% of participants, respectively, and missing covariate data were each <3%, leading to the exclusion of < 5% of the eligible cohort.

### Exposure assessment

Short-term exposure to particulate matter ≤ 10 µm (PM₁₀) was estimated for each participant over the day preceding the day of recruitment, under the hypothesis that short-term environmental stimuli may influence epigenetic and inflammatory markers. Daily PM₁₀ data were collected from the Air Quality Monitoring Network of ARPA Lombardia (Regional Environmental Protection Agency), using Fixed monitoring stations, providing direct daily PM₁₀ measurements for each subject’s residential address. Short-term PM₁₀ exposure was defined as the average concentration on the day of blood sampling and the previous day (lag 0–1), assigned to each participant based on residential address. This exposure window was selected a priori based on prior analyses in the same SPHERE cohort [19], in which the relationship between PM and fibrinogen was evaluated across multiple lag structures and lag 0–1 was identified as the most consistent and biologically plausible interval. Moreover, PM₁₀ was selected as the primary exposure metric because high-quality daily measurements with complete temporal coverage were available for the entire study period from the regional environmental protection agency (ARPA Lombardia). By contrast, PM₂.₅ data were not consistently available for all participants, which would have markedly reduced the sample size and statistical power. However, the two measures PM₁₀ and PM₂.₅ from previous studies are expected to be highly correlated.

Exposure data were assigned using ArcGIS^®^ software (Esri). For each participant, we extracted the daily PM₁₀ values from the nearest monitor to the residential address. Meteorological variables (temperature and relative humidity) were obtained from ARPA stations across the region (233 for temperature, 163 for humidity). Apparent temperature was calculated according to previously validated methods [46]. Further technical details on exposure assessment methodology have been described elsewhere [47].

### Sample collection and DNA methylation analysis

Whole blood (7 ml) was collected from each participant using EDTA tubes and immediately stored at −80°C. Genomic DNA was then extracted from whole blood using the Wizard Genomic DNA Purification Kit (Promega, Madison, WI, USA), following the manufacturer’s instructions. DNA concentration was measured and adjusted to 25 ng/µl. A total of 500 ng of DNA per sample was used for bisulfite conversion. Bisulfite treatment was performed using the EZ DNA Methylation-Gold Kit (Zymo Research, Orange, CA, USA), according to the manufacturer’s protocol. Final DNA elution was carried out in 200 µl of M-Elution Buffer provided with the kit.

To evaluate methylation of LINE-1 repetitive elements, which represent a substantial portion of the human genome and reflect the epigenetic state of interspersed repeats, methylation levels at consensus LINE-1 sequences were quantified by pyrosequencing, following previously published procedures with minor modifications [21]. Polymerase chain reaction (PCR) amplification was performed in a 50 µl reaction volume containing 25 µl of GoTaq Green Master Mix (Promega), 10 pmol of each primer, and 50 ng of bisulfite-treated genomic DNA. The forward primer sequence was 5′-TTTTGAGTTAGGTGTGGGATA-3′, and the reverse primer was biotinylated at the 5′ end: 5′-biotin-AAAATCAAAAAATTCCCTTTC-3′. The PCR cycling protocol consisted of an initial denaturation at 95°C for 30 s, followed by 35 cycles of 50°C for 30 s and 72°C for 30 s. The biotin-labeled PCR products were immobilized on Streptavidin Sepharose HP beads (Amersham Biosciences, Uppsala, Sweden) and processed using the Pyrosequencing Vacuum Prep Tool (Pyrosequencing, Inc., Westborough, MA, USA) to isolate single-stranded DNA. The sequencing primer, 5′-AGTTAGGTGTGGATATAGT-3′, was then annealed (0.3 µM final concentration), and pyrosequencing was carried out using the PyroMark MD System (Pyrosequencing, Inc.).

Methylation percentages were calculated as the ratio of methylated cytosines to the total number of cytosines at the interrogated CpG sites.

### Conceptual framework and causal diagram

The analytical framework was based on the EI model, in which repetitive DNA methylation levels are the result of cumulative environmental and endogenous influences and can modulate the organism’s response to acute stimuli. In this study, LINE-1 methylation was considered as an environment-shaped baseline biomarker potentially modifying the short-term effect of PM₁₀ on fibrinogen levels. While PM₁₀ may exert a direct, albeit modest, effect on LINE-1 methylation, this biomarker is largely determined by the integrated exposome and thus can act as a systemic modulator of inflammatory reactivity.

**Figure 3.**
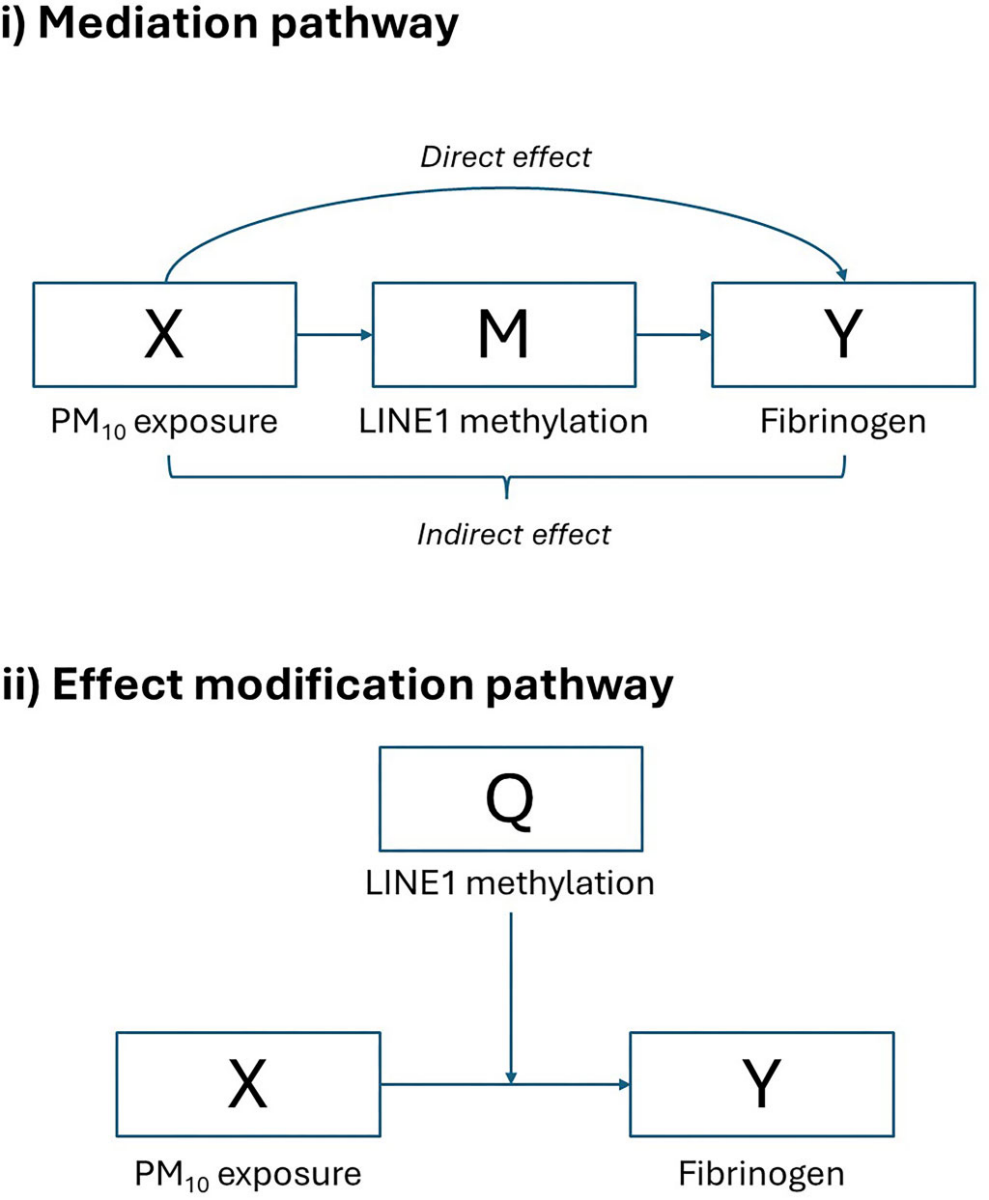
Directed Acyclic Graph (DAG) of the hypothesized relationships among PM₁₀ exposure, LINE-1 methylation, and fibrinogen. The DAG illustrates two potential pathways: (i) a mediation pathway, where PM₁₀ directly alters LINE-1 methylation, which in turn affects fibrinogen levels; and (ii) an effect modification pathway, where LINE-1 methylation, shaped by cumulative life-course exposures, genetic background, and lifestyle factors, modulates the association between PM₁₀ and fibrinogen. According to the Epigenetic Intelligence (EI) framework, LINE-1 methylation is conceptualized as a systemic modulator or buffering factor, rather than a simple mediator of acute pollutant effects.

To make this conceptual structure explicit, we developed a directed acyclic graph (DAG, Fig. 3) illustrating the hypothesized relationships among PM₁₀ exposure, LINE-1 methylation, and fibrinogen, along with measured confounders and covariates. The diagram depicts both the primary pathway of interest, effect modification by LINE-1, and the plausible but secondary mediation pathway.

### Statistical analysis

We first performed descriptive analyses to characterize the distribution of the main study variables, including fibrinogen levels, LINE-1 methylation, and PM₁₀ exposure. Continuous variables were summarized using medians and interquartile ranges (means and standard deviations for symmetric distributions), while categorical variables were reported as counts and percentages. Graphical methods such as histograms, boxplots, and dotplots were employed to assess variability and detect potential outliers. Pairwise relationships between key variables were explored using scatterplots and correlation coefficients (Pearson or Spearman, as appropriate), to identify potential collinearity and inform model specification. These preliminary analyses guided the selection of statistical models by revealing distributional features, non-linear trends, and possible interaction structures.

For downstream analyses, LINE-1 methylation percentage values were logit-transformed to linearize relationships and stabilize variance. We used a robust GAMs with a scaled t-distribution family to model the dependence of fibrinogen from PM₁₀, to improve robustness to deviations from normality and to reduce the influence of extreme fibrinogen values. This distribution has heavier tails than the Gaussian, allowing for more stable parameter estimates in the presence of outliers or high-leverage observations. To assess the hypothesized effect modification, the main model included a tensor product smooth interaction term between PM₁₀ and LINE1 methylation [te(PM_10_, LINE1)], enabling flexible modeling of non-linear interactions.

All models were adjusted for potential confounders known to influence inflammation or DNA methylation, including age (continuous), sex, and menopausal status (combined as a 3-level categorical variable: males, premenopausal females, postmenopausal females), body mass index (BMI, continuous), smoking status (current/former/never), monocyte counts (continuous), and CRP levels (continuous).

Model fit was assessed by explained deviance and adjusted R². Competing models (with and without interaction) were compared using Akaike Information Criterion and F-tests for nested models. We also applied MARS as a complementary approach to identify key interaction terms and assess relative variable importance.

All analyses were conducted using R version 4.4.3 (2025–02-28), with the mgcv and earth packages.

## Supplementary Material

dvaf028_Supplemental_File

## Data Availability

Not applicable.
