# Supplementary material for: Redundancy, noise, and plasticity: repetitive DNA as an epigenetic intelligence backbone of inflammatory regulation
Source: Environ Epigenet. 2025 Oct 22;11(1):dvaf028. doi: 10.1093/eep/dvaf028 (PMC12587764; doi:10.1093/eep/dvaf028)
Supplement: dvaf028_Supplemental_File [file dvaf028_supplemental_file.docx]

# Supplementary Table S1. Sensitivity analyses for the association between PM10, LINE-1 methylation, and fibrinogen levels, considering season and temperature.

| **Variable** | **Base model Estimate (SE), p** | **Adjusted model (season + temp) Estimate (SE), p** |
| --- | --- | --- |
| PM_10_ | 1.057 (0.395), 0.0075 | 0.969 (0.394), 0.014 |
| logit_LINE1 | 45.35 (16.95), 0.0075 | 41.98 (16.99), 0.013 |
| PM_10_ × LINE1 interaction | -0.842 (0.362), 0.020 | -0.785 (0.363), 0.030 |
| Age | 0.163 (0.117), 0.164 | 0.171 (0.117), 0.143 |
| Sex: Female (non-menopausal) | 12.80 (3.56), <0.001 | 13.30 (3.53), <0.001 |
| Sex: Female (menopausal) | 23.69 (3.31), <0.001 | 23.92 (3.30), <0.001 |
| Season: Spring | – | -13.58 (4.40), 0.002 |
| Season: Summer | – | -9.77 (6.63), 0.141 |
| Season: Autumn | – | 1.12 (4.28), 0.795 |
| s(BMI) | edf=1.00, p<0.001 | edf=1.00, p<0.001 |
| s(CRP) | edf=3.50, p<0.001 | edf=3.50, p<0.001 |
| s(Monocytes) | edf=1.99, p<0.001 | edf=1.94, p=0.002 |
| s(Temperature) | – | edf=1.14, p=0.201 |

## Model fit summary

| **Metric** | **Base model** | **Adjusted model** |
| --- | --- | --- |
| Adjusted R² | 0.392 | 0.399 |
| Deviance explained | 32.8% | 33.5% |
| N | 1527 | 1526 |
